# Scanning MEMS Mirror for High Definition and High Frame Rate Lissajous Patterns

**DOI:** 10.3390/mi10010067

**Published:** 2019-01-18

**Authors:** Yeong-Hyeon Seo, Kyungmin Hwang, Hyunwoo Kim, Ki-Hun Jeong

**Affiliations:** 1 Department of Bio and Brain Engineering, KAIST, Daejeon 34141, Korea; yseo@kaist.ac.kr (Y.-H.S.); k.hwang@kaist.ac.kr (K.H.); hkim151@kaist.ac.kr (H.K.); 2 KAIST Institute for Health Science and Technology, Daejeon 34141, Korea

**Keywords:** MEMS mirror, Lissajous scanning, pseudo-resonant, sensing, imaging, display

## Abstract

Scanning MEMS (micro-electro-mechanical system) mirrors are attractive given their potential use in a diverse array of laser scanning display and imaging applications. Here we report on an electrostatic MEMS mirror for high definition and high frame rate (HDHF) Lissajous scanning. The MEMS mirror comprised a low Q-factor inner mirror and frame mirror, which provided two-dimensional scanning at two similar resonant scanning frequencies with high mechanical stability. The low Q inner mirror enabled a broad frequency selection range. The high definition and high frame rate (HDHF) Lissajous scanning of the MEMS mirror was achieved by selecting a set of scanning frequencies near its resonance with a high greatest common divisor (GCD) and a high total lobe number. The MEMS mirror had resonant scanning frequencies at 5402 Hz and 6702 Hz in *x* and *y* directions, respectively. The selected pseudo-resonant frequencies of 5450 Hz and 6700 Hz for HDHF scanning provided 50 frames per second with 94% fill factor in 256 × 256 pixels. This Lissajous MEMS mirror could be utilized for assorted HDHF laser scanning imaging and display applications.

## 1. Introduction

Microscanners play a vital role in various low-power and compact scanning applications, including in display [1,2,3,4], sensing [5,6,7,8], and biomedical imaging [9,10,11,12,13,14,15,16]. In particular, resonant MEMS (micro-electro-mechanical system) mirrors provide a focus beam with a small size and high energy efficiency at any distance [17,18,19,20] and the monolithic fabrication facilitates low-cost commercialization [21,22]. Unlike raster scanning MEMS mirrors, Lissajous MEMS mirrors operate at high scanning frequencies in both axes and also offer simple fabrication [3,23], high mechanical stability [17,24], and uniform scanning quality [21,25]. However, in MEMS mirrors, there is still a trade-off between the frame rate (FR) and the fill factor (FF) for high quality laser scanning. 

This trade-off relationship restricts the implementation of high definition and high frame rate (HDHF) Lissajous scanning. The FF increases as the ratio of two scanning frequencies becomes more complex, whereas the pattern repeat rate is lessened. Full-repeated Lissajous scanning often has a low frame rate, which is determined by the pattern repeat rate. In contrast, non-repeated Lissajous scanning provides a higher frame rate than the pattern repeat rate [26]. However, non-repeated scanning exacerbates the trade-off relationship between the FF and the FR. Besides, the frame rate can be determined by the ratio of two scanning frequencies [2,3], but the frame rate is usually a non-integer. The resonant frequency of Lissajous MEMS mirrors is highly dependent on the physical dimension and, thus, an integer frame rate is barely obtained due to the microfabrication tolerance. Furthermore, some technical artifacts, such as flickering phenomenon, occur [2]. Recently, the frequency selection rule for HDHF Lissajous scanning has been reported to overcome the trade-off relationship [27]. The FR increases the greatest common divisor (GCD), while the FF increases with the total lobe number, i.e., divided by the sum of two scanning frequencies as the GCD of the two scanning frequencies. The frequency selection should satisfy a high GCD and high total lobe number results in HDHF Lissajous scanning. HDHF Lissajous scanning with 10 frames per seconds and 92% fill factor was demonstrated with a 1 kHz scanning frequency. HDHF Lissajous scanning has been successfully implemented in PZT (piezo-tube) fiber scanners; however, it has not yet been achieved with MEMS mirrors. PZT fiber scanners have been widely used for endomicroscopic applications [16,28,29]; however, they still have some technical limitations including a high cost and the need for further miniaturization. The conventional Lissajous mirror has a high Q-factor, which substantially changes the scanning amplitude depending on the scanning frequency. For this reason, the conv-entional Lissajous mirror still has technical limitations for HDHF Lissajous scanning applications. 

**Figure 1:**
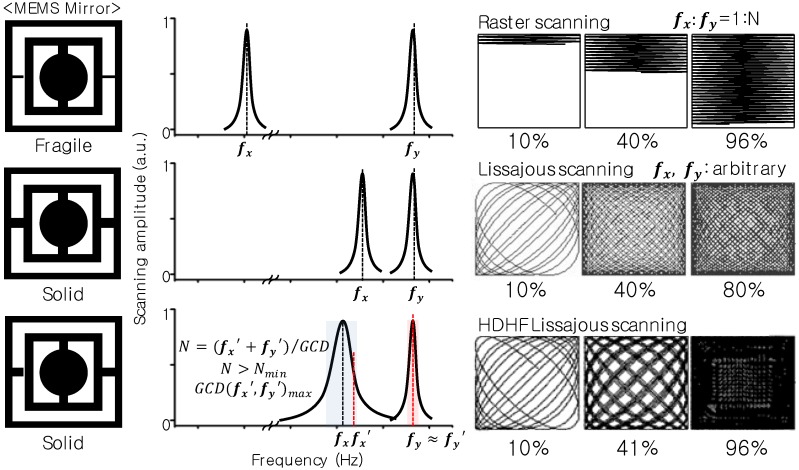
A schematic illustration of the high definition, high frame rate (HDHF) Lissajous MEMS mirror. A conventional raster MEMS mirror provides two-dimensional (2D) scanning with a ratio of two frequencies of 1:N and a torsional micromirror usually requires a flexible micro-spring. Unlike raster scanning MEMS mirrors, conventional Lissajous MEMS mirrors have high mechanical stability. Conventional Lissajous mirrors also have a high Q factor, which derives a substantial change of the scanning amplitude depending on the scanning frequency. In contrast, the HDHF Lissajous MEMS mirror features an inner mirror with a low Q-factor, which allowed a broad selection range of scanning frequencies. The selection of scanning frequencies at pseudo-resonance with a high greatest common divisor (GCD) and a high total lobe number (*N*, ($f_{x}$ + $f_{y}$)/GCD), which is larger than the minimum total lobe number ($N_{min}$) for the targeted FF, enabled HDHF Lissajous scanning. Since the designed MEMS mirror is for 2D laser scanning, biaxial scanning frequencies had to be selected. Note that $f_{x}$ and $f_{y}$ infer the resonant frequencies of the inner mirror and the frame mirror, respectively. In addition, biaxial scanning frequencies are defined as $f_{x}$’ and $f_{y}$’, which infer the selected freque-ncies of the inner mirror and frame mirror, respectively, determined by the frequency selection rule.

Here we report an electrostatic MEMS mirror capable of high definition and high frame rate (HDHF) Lissajous scanning. Figure 1 indicates a schematic illustration of the high definition and high frame rate (HDHF) Lissajous MEMS scanner. The HDHF Lissajous MEMS mirror features an inner mirror with a low Q-factor, which allowed a broad selection range of scanning frequencies. A low Q-factor of the inner mirror was realized by using a thin and short torsion bar. The selection of scanning frequencies at pseudo-resonance with a high GCD and high total lobe number (*N*, ($f_{x}$ + $f_{y}$)/GCD), which is larger than the minimum total lobe number ($N_{min}$) for the targeted FF, allowed HDHF Lissajous scanning. Note that $f_{x}$ and $f_{y}$ infer the resonant frequencies of the inner mirror and frame mirror, respectively, whilst $f_{x}$’ and $f_{y}$’ infer the selected scanning frequencies of the inner mirror and frame mirror, respectively, as determined by the frequency selection rule.

## 2. Device Fabrication and Characterization

**Figure 2:**
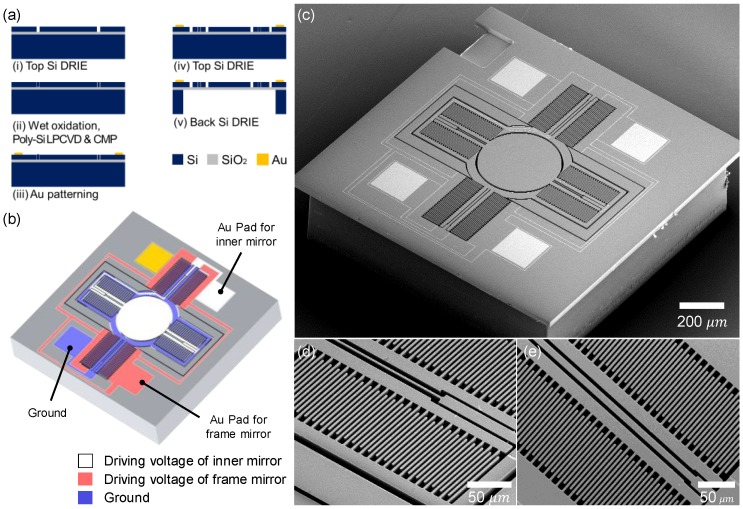
Microfabrication procedure and SEM images of HDHF Lissajous MEMS mirror. (**a**) Microfabrication procedure. The MEMS mirror was fabricated using a 6-inch SOI wafer with high conductivity. (**b**) A schematic of the electrical layout of the MEMS mirror. (**c**) Top SEM image of the microfabricated HDHF Lissajous MEMS mirror (scale bar: 200 μm). The physical dimensions of the MEMS mirror were 1.2 × 1.2 × 0.43 mm^3^. (**d**–**e**) Perspective SEM images of the comb drives of the inner mirror and the frame mirror, respectively (scale bar: 50 μm). The widths of the torsion bar were 2.8 μm and 8.8 μm in the inner mirror and the frame mirror, respectively.

The microfabrication procedure of the Lissajous MEMS mirror is described in Figure 2a. A MEMS mirror was fabricated using a 6-inch SOI wafer (silicon-on-insulator wafer, top Si: 30 μm, buried oxide (BOX) layer: 2 μm, bottom Si: 400 μm) with high conductivity (resistivity: 0.01–0.02 Ω·cm). Firstly, the top silicon layer was defined by deep reactive ion etching (DRIE) and refilled with silicon dioxide through wet oxidation and poly-silicon by low pressure chemical vapor deposition (LPCVD). The front side was flattened using chemical mechanical polishing (CMP), followed by the thermal evaporation of a 200-Å-thick titanium and 1000-Å-thick gold film. The Au electrode pads were patterned with a wet-etch process. The top silicon layer and bottom silicon layers were etched down to define the MEMS mirrors with a backside opening using DRIE. A buried oxide layer of the opening area was removed in buffered oxide etchant (BOE) to release the MEMS mirror. The remaining photoresist layers were clearly stripped out by using oxygen plasma. Individual MEMS mirrors were completely detached from the SOI wafer using the fused-tether method with Y-shape tethers with a width of 4 μm [30]. Figure 2b shows a scheme of the electrical layout of the MEMS mirror. Gray, red, and blue indicate the driving voltage of the inner mirror, the driving voltage of the frame mirror, and the ground, respectively. Figure 2c indicates a top SEM image of the fabricated Lissajous MEMS mirror (scale bar: 200 μm). The physical dimensions of the MEMS mirror were 1.2 × 1.2 × 0.43 mm^3^. Figure 2d,e show the perspective SEM images of the comb drives of the inner mirror and the frame mirror, respectively. The widths of the torsion bar were 2.8 μm and 8.8 μm in the inner mirror and the frame mirror, respectively. The Q-factor was determined by the flexure width, height, and length of the MEMS mirror. A thin torsion bar provided a low Q-factor for the inner mirror, which made a broad frequency tuning range [31]. The effective stiffness and the resonant frequencies of the Lissajous MEMS mirror were calculated using finite element analysis (COMSOL Multi-physics^®^ ver. 5.3). The MEMS mirror provided a high yield (95% yield, 2000 mirrors in a 6-inch wafer).

## 3. Scanning Frequency Selection for HDHF Lissajous Scanning

**Figure 3:**
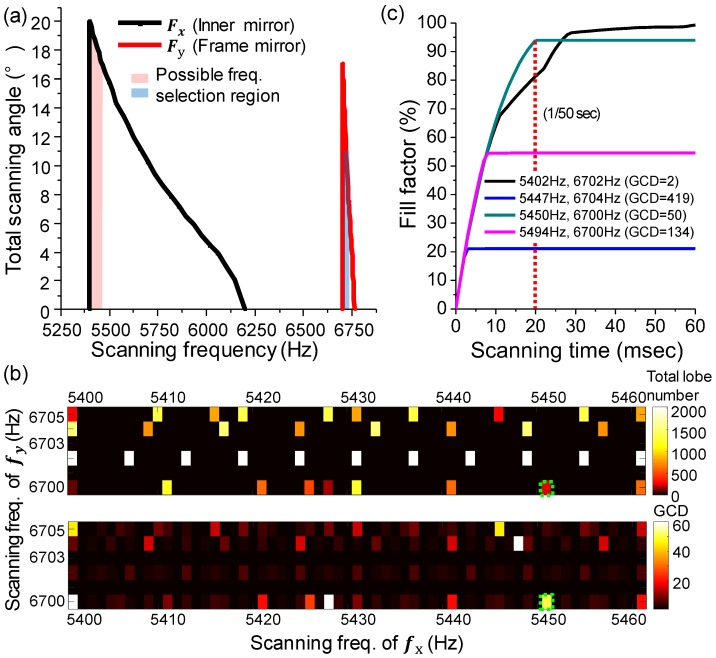
Scanning properties and scanning frequency selection for HDHF Lissajous scanning. (**a**) Frequency response of the HDHF Lissajous MEMS mirror. The MEMS mirror has resonant frequencies at 5402 Hz and 6702 Hz in the inner mirror and the frame mirror, respectively. The frequency tuning range should be larger than the greatest common divisor (GCD) frame rate. The inner mirror features a low Q-factor (Q = 18) for frequency selection. (**b**) Color maps of the GCD and total lobe number for selecting scanning frequency along biaxial frequency domain. High GCD and high total lobe number allow high definition and high frame rate Lissajous scanning. The selected frequency sets for HDHF Lissajous scanning should satisfy the requirements for both a high GCD and high lobe number. For instance, based on the frequency selection rule, the fill factor was over 85% at 256 × 256 pixel resolution, while the total lobe number was 237 or more. Frequency sets where the total lobe number was greater than 237 in the first color map were selected. Next, in the second color map, a frequency set with the higher GCD value was selected from the previously selected frequency set. The scanning frequencies were determined as 5450 Hz and 6700 Hz (GCD = 50, total lobe number = 243) for HDHF Lissajous scanning. HDHF frequency set was selected within the range of 1% of the resonant frequency. (**c**) Calculated fill factor (FF) of the MEMS mirror along the scanning time. The fill factor initially increased with time; however, the maximum FF and the convergence time varied with the set of selected scanning frequencies. The scanning frequencies of 5450 Hz and 6700 Hz provide a single Lissajous figure of 94% in FF at every 1/50 s.

Figure 3a indicates the frequency response of the HDHF Lissajous MEMS mirror. The frequency response was obtained by measuring the scanning angle of the MEMS mirror depending on the operation frequency. The MEMS mirror had a resonant frequency at 5402 Hz and 6702 Hz in the inner mirror and the frame mirror, respectively. The total scanning angles were 20° and 18° with 40 $V_{pp}$ operation voltages in the inner mirror and the frame mirror, respectively. The inner mirror featured a low Q-factor (Q = 18) for a broad band selection of scanning frequency. The Q-factor is calculated as Q = $f_{r}/\Delta f_{fwhm}$ ($f_{r}$: resonant frequency, $\Delta f_{fwhm}$: the full width at half maximum). The scanning frequencies were selected at the frequencies with a 0.7 dB bandwidth. Figure 3b shows the color maps of the GCD and the total lobe number with the different scanning frequencies. A high GCD and high lobe number play a significant role when selecting the frequency sets for HDHF Lissajous scanning. In order to acquire a fill factor over 85% at 256 × 256 pixel resolution, the required total lobe number based on the frequency selection rule should be greater than 237. The first color map in Figure 3b selects the frequency sets where the total lobe number is greater than 237. The second color map in Figure 3b selects the frequency set with the highest GCD value among the selected frequency sets in the first color map. The scanning frequencies were determined as 5450 Hz and 6700 Hz (GCD = 50, total lobe number = 243) for HDHF Lissajous scanning. Figure 3c shows a calculated fill factor (FF) along the scanning time. The convergence time and the maximum fill factor were apparently different as the scanning frequencies changed, while the FF increased with time. The selected scanning frequencies of 5450 Hz and 6700 Hz provided HDHF scanning (94% fill factor at 1/50 s).

**Figure 4:**
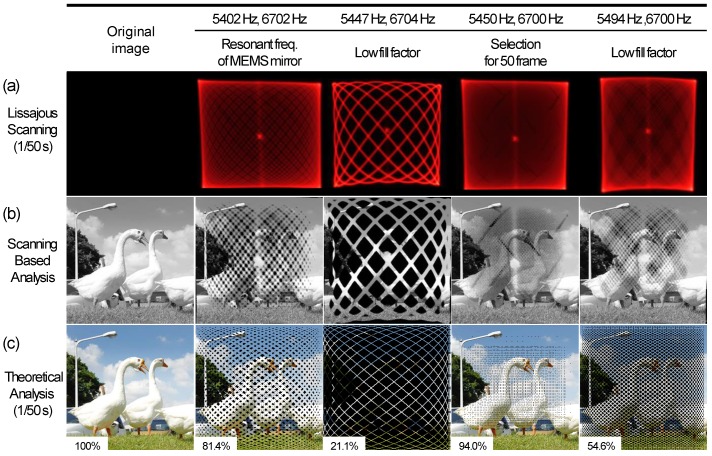
Lissajous scanning patterns and theoretical trajectory depending on different scanning frequencies. (**a**) Optical images of the Lissajous scanning patterns depending on different scanning frequencies (1/50 s). (**b**) Pattern-projected image of the geese. The Lissajous scanning pattern of (a) was projected onto the image of geese. (**c**) Theoretical analysis of Lissajous laser trajectory depending on different scanning frequencies at 1/50 s. Simulation was conducted using MATLAB R2017a and the source image had a 256 × 256 pixel resolution. Compared to resonance scanning, scanning at the selected frequencies of 5450 Hz and 6700 Hz provided a high fill factor with a high frame rate.

Figure 4a shows optical images of Lissajous scanning patterns at different sets of scanning frequencies (1/50 s). Scanning patterns were obtained at scanning frequencies of 5402 Hz/6702 Hz, 5447 Hz/6704 Hz, 5450 Hz/6700 Hz, and 5494 Hz/6700 Hz, respectively. Scanning at the selected frequencies of 5450 Hz and 6700 Hz provided a high fill factor, compared to other frequencies, including the resonant frequency. Figure 4b shows a pattern-projected image of the geese, wherein the Lissajous scanning pattern of Figure 4a was projected onto the image of geese. Figure 4c shows a theoretical analysis of the Lissajous laser projected image at 1/50 s. The simulation was conducted using MATLAB R2017a. The Lissajous pattern with two scanning frequencies was tracked through 256 × 256 pixels for 1/50 s and then overlapped with the image. The Lissajous scan trajectory gradually filled with time, and the fill factor at the selected frequencies of 5450 Hz and 6700 Hz reached up to 94% in 1/50 s. Compared to the resonance scanning, pseudo-resonant scanning at the selected frequencies provided a high fill factor with a high frame rate. In addition, Table 1 and Table 2 show assorted sets of HDHF scanning for 256 × 256 and 1280 × 720 pixel resolutions, respectively. The HDHF MEMS mirror clearly provides a wide tuning range, as well as various frame rates with a high fill factor. 

## 4. Summary

In summary, we have successfully demonstrated HDHF Lissajous MEMS mirrors. The Lissajous MEMS mirror had a low Q-factor for the inner mirror for a broad frequency tuning range, as well as similar resonant frequencies in the inner mirror and the frame mirror, with high mechanical stability. The MEMS mirror had resonant frequencies at 5402 Hz and 6702 Hz in the inner mirror and the frame mirror, respectively. The total scanning angles were 20° and 18° with 40 $V_{pp}$ operation voltages in the inner mirror and the frame mirror, respectively. High definition and high frame rate (HDHF) Lissajous scanning was successfully realized by applying the scanning frequency selection. The controlled Lissajous MEMS mirror provided a 94% fill factor at 50 frames per second for 256 × 256 pixels. In addition, diverse sets of HDHF scanning were demonstrated for potential use in various applications. HDHF Lissajous MEMS mirrors can be utilized for assorted laser scanning-based imaging and display applications.

## Figures and Tables

**Table 1:** A set of scanning frequencies with different frame rates and fill factors for 256 × 256 pixels.

Frequency (Hz)	5408/6704	5420/6700	5425/6700	5450/6700	5427/6700
Greatest common divisor (GCD) (frame rate (FR))	16	20	25	50	67
Fill factor (%)	100	98	100	94	84

**Table 2:** A set of scanning frequencies with different frame rates and fill factors for 1280 × 720 pixels.

Frequency (Hz)	5400/6702	5400/6708	5410/6700	5408/6704	5420/6700
GCD (FR)	6	8	10	16	20
Fill factor (%)	100	100	100	90	52
